# Spectral Pattern Classification in Lidar Data for Rock Identification in Outcrops

**DOI:** 10.1155/2014/539029

**Published:** 2014-02-18

**Authors:** Leonardo Campos Inocencio, Mauricio Roberto Veronez, Francisco Manoel Wohnrath Tognoli, Marcelo Kehl de Souza, Reginaldo Macedônio da Silva, Luiz Gonzaga Jr, César Leonardo Blum Silveira

**Affiliations:** ^1^VIZLab, Advanced Visualization Laboratory, UNISINOS, 93022-000 São Leopoldo, RS, Brazil; ^2^PPGEO, Graduate Program on Geology, UNISINOS, 93022-000 São Leopoldo, RS, Brazil; ^3^PIPCA, Applied Computer Science Graduate Program, UNISINOS, 93022-000 São Leopoldo, RS, Brazil; ^4^V3D, Studios & Ficta Mobile Technologies, 93022-000 São Leopoldo, RS, Brazil

## Abstract

The present study aimed to develop and implement a method for detection and classification of spectral signatures in point clouds obtained from terrestrial laser scanner in order to identify the presence of different rocks in outcrops and to generate a digital outcrop model. To achieve this objective, a software based on cluster analysis was created, named K-Clouds. This software was developed through a partnership between UNISINOS and the company V3D. This tool was designed to begin with an analysis and interpretation of a histogram from a point cloud of the outcrop and subsequently indication of a number of classes provided by the user, to process the intensity return values. This classified information can then be interpreted by geologists, to provide a better understanding and identification from the existing rocks in the outcrop. Beyond the detection of different rocks, this work was able to detect small changes in the physical-chemical characteristics of the rocks, as they were caused by weathering or compositional changes.

## 1. Introduction

Geology, like other sciences, enjoys technological advances to increasingly develop their methods and enhance the expertise of their field of study. New equipment and methods are in constant development to support these applications and from all of this equipment and systems developed in recent years, the laser scanning and profiling has been consolidated as one of the most effective technology for geospatial data acquisition.

The automated data collection has expanded rapidly in recent years in line with the technological advances made in the areas of surveying and mapping [1]. The use of such equipment for geological studies was a natural and logical step, considering that the collection and use of data from outcrops are the main source for the works of surface geology.

The digital representation in geological studies evolved from LANDSAT satellite imagery, digital terrain models in conjunction with image processing (i.e., [2]), and aerial photos interpretation techniques combined with the Global Navigation Satellite System (GNSS) (i.e., [3]). Over the past decade the use of digital mapping technologies have increased, in particular with the use of terrestrial laser scanner surveying and other systems integrated with satellite navigation and geographic information [4–6] which are replaced with many advantages of photographic mosaics, routinely used in the interpretation of large outcrops.

The laser scanning and profiling systems, also known as terrestrial laser scanner (TLS), have some characteristics that apply significantly for geological purposes, such as the fast acquisition of data of a particular outcrop in distances that can reach 2 km, the high definition of the spatial resolution, that is, the spacing between the points collected at a certain distance and high degree of accuracy of the survey.

The use of TLS in outcrop studies is expanding due to the ease of acquisition of georeferenced data in an accurate, fast, and automated way. Its use for this purpose began about a decade ago [7], but only in recent years the number of scientific papers has increased significantly. Topics of interest are varied, with emphasis on the methodological approaches [1, 8–11], reservoirs analogues studies [12–19] of fractured rocks [20–23], slope stability [24], erosion rates [25], synthetic seismic models [26], geological heritage [27], determining the direction of basaltic lava flow [28] geology education [29, 30], and outcrop interpretation [31].

The equipment available on the market is able to conduct the survey and collection of thousands (time of flight) up to millions (phase) of points per second and some have the ability to record the intensity of the pulse return for each position of the laser X/Y/Z [8]. The intensity of the return pulse from the laser and the equipment range are directly related to factors that affect the working of the system. Among these factors we can mention the power of the emitted pulse and the reflectivity of the targets.

The relation between the reflectivity of the targets and the intensity of the laser beam emitted is directly proportional to the range of the Laser Scanner. If a target has 10% reflectivity, the equipment should be closer than a target that has 90% reflectivity [32].

As other active remote sensing systems, the TLS has only one wavelength, as the intensity of the return pulse is based on the interaction of the laser's specific wavelength with the target. This interaction may be analyzed using interferometry techniques, which are the study of phenomena caused by the interfering within the light waves, which, in this case, are the interaction between the laser wavelength and the physical-chemical characteristics of the targets [33].

As we use TLS to survey an outcrop, we not only collect their spatial characteristics, such as features and volumes, but we also obtain additional data that may allow a greater understanding of the outcrop.

In most cases, each TLS equipment generates its own file format for archiving of the collected data. However, these files have similarities concerning the information stored. These files record the spatial position of the points (X, Y, and Z), the intensity of the laser pulse that returns from the target (i) and the RGB color pattern obtained from the pictures recorded by the internal camera. The output is a txt file that provides the sequence X, Y, Z, I, R, G, B.

The spatial information is commonly used for volume calculations and identification of geometric features and the RGB data assist in identifying visual features and provide a better understanding of the objects surveyed with the TLS, but when it comes to the intensity values of the return laser pulse, there is a lack of specific applications designed to transform this data in information, which can detect variations in the physical-chemical characteristics of the targets.

Thus, the main objective of this study was to develop a method for classification of spectral patterns in LIDAR (light detect and range) data for the identification of rocks in outcrops with distinct lithology.

The validation of this method occurred through the use of a cluster analysis algorithm known as *k*-means. The choice of this algorithm was given by the obtained separation of the spectral responses of rocks with different characteristics, both chemical and/or physical. This allowed us to classify the patterns of spectral response and thus identify rocks and features present in the outcrops. This classifier tool will be incorporated in the software Mountain View (partnership V3D/UNISINOS), which allows the user view, edits and processes data from TLS, and the main application is focused on Digital Outcrop Modeling (DOM).

## 2. Terrestrial Laser Scanner

The Laser Scanning and profiling systems, in this case terrestrial, basically consist of a measuring instrument based on laser that can measure vertical angles, horizontal angles, and distances with a high standard of accuracy and speed by means of a mobile mirrors or prisms system that allow the mapping of topographical features on targets.

**Figure 1:**
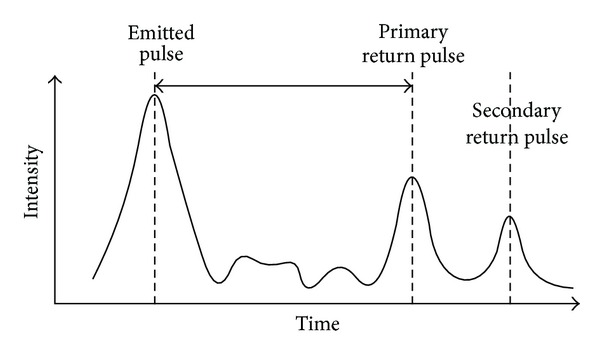
Time of flight measurement system operation principle, where the distance is calculated dividing by 2 the time elapsed between the emitted pulse and the primary or secondary return pulse. Modified from San Jose Alonso et al. [34].

These systems have different methods for measure distances, and the time of flight (TOF) (Figure 1) and phase shift (PS) are the most widespread. In the Time of Flight method the instrument sends a high-intensity laser pulse toward the target and measure the time between sending and receiving the pulse returns and divides the value by 2 to calculate the distance.

**Figure 2:**
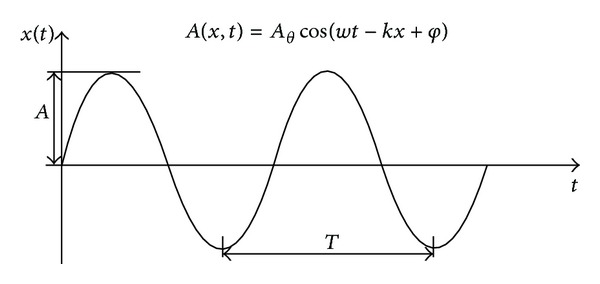
Phase shift measurement system operation principle, where the distance is calculated comparing the phase difference between the emitted pulse and the return pulse. Modified from San Jose Alonso et al. [34].

In the phase shift method, the instrument emanates a laser pulse fitted within a harmonic wave and the distance is calculated by comparing the difference of the transmitted and received waves [35] (Figure 2).

Both methods of data acquisition have strengths and weaknesses due to the method applied. The equipment that uses the phase shift technology tends to collect more points per second at the same time interval, millions of points per second, while the time of flight collects thousands of points per second. However, the phase shift scanners have a reduced range that varies between 80 to 120 meters, against up to two kilometers in some time of flight equipment. Another element to consider is that the operation of phase shift equipment in conditions of high insolation can reduce the range of the equipment.

All the elements quoted above must be taken into consideration when selecting the most suitable technology to perform the work, in which case, Digital Outcrop Modeling.

## 3. Classification

The term classification is defined as an actual or ideal arrangement, relating what is similar and separating what is different, having as main objectives to maintain and build knowledge and analyze and relate the structure of certain phenomena [36]. If we apply this definition to geology, specifically in outcrops classification, it becomes clear that the first and main elements to be classified and identified are the rocks on site. This information is intended to expand the knowledge of the geologists regarding the elements present in the outcrop, allowing a greater understanding of existing processes and materials.

In digital image processing, different classifiers are well established, as they are applied to specific purposes or applied to general purpose or even allowing their use for applications in a classification tool that is both specific and generic.

Works such as those developed by Brodu and Lague [37] and Franceschi et al. [38] are intended to implement different methods of classification in TLS data for extracting information. These classification tools allow a fast processing of database containing thousand to million points, acquired by TLS.

In the various methods for data classification, data clustering, also called cluster analysis, segmentation analysis, taxonomic analysis, or unsupervised classification, is a method of creating groups of objects that at first performs the grouping of all objects that have a high degree of similarity, always ensuring that very different objects do not belong to the same group (cluster).

The data clustering techniques are often confused with classification, in which objects are assigned to predefined classes, but in data clustering the classes must also be defined [39]. Compared with other types of sorting algorithms, clusters based algorithms are very efficient for classification of large databases and multidimensional data [39].

The classifier *k*-means, first described by MacQueen in 1967, is a cluster classifier that performs a process of partitioning of a population “*N*” into “*k*” classes. These partitions represent satisfactorily the internal variability that occurred within each class. Besides the described above, the *k*-means classifier is easily programmable and computationally economical, being able to process large volumes of data in applications such as grouping by similarity, predicting nonlinear approximation of multivariate distributions, and nonparametric tests, among others [40]. These features become suitable for use in applications related to geology, where the study areas can be located inside a few meters or along several kilometers. This scale variability, together with the type of data separation carried out by the classifier *k*-means, which is capable of displaying discrete patterns of variation, allows a proper understanding of the data.

## 4. Study Areas

**Figure 3:**
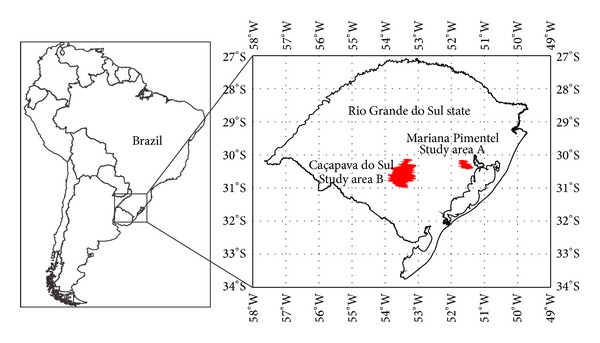
Localization of the study areas in Mariana Pimentel (A) and Caçapava do Sul (B).

For the present work two study areas were selected, in the cities of Mariana Pimentel and Caçapava do Sul, both in Rio Grande do Sul state (Figure 3). Beyond the study areas, initial tests were also carried out on rock samples to define the work method to be used in the Laboratory of Remote Sensing and Digital Cartography (LASERCA) in Unisinos.

**Figure 4:**
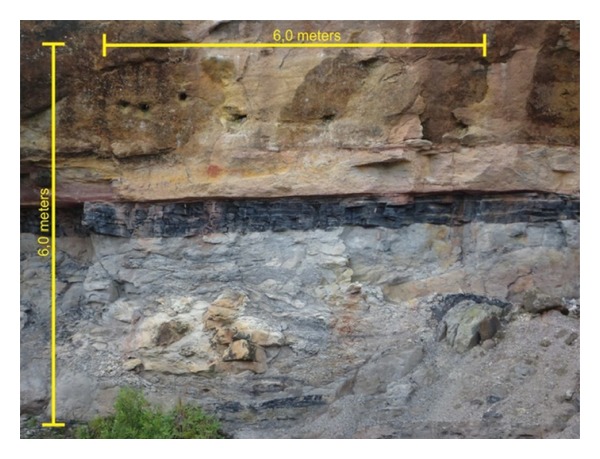
Study area A, Morro do Papaléo outcrop, Rio Bonito Formation. From the base to top we can identify the diamictite (light gray, bottom part), the carbonaceous pelite (black, in the middle part), and the sandstone (light and medium brown, top part).

The study area A is located in the municipality of Mariana Pimentel, approximately 80 km from Porto Alegre with access from the highway BR-116 and after municipal and private roads. In this place the outcrop called “Morro do Papaléo” is located at coordinates: Latitude 30°18′29,56′′ S and Longitude 51°38′35,26′′ W. Gr. (SIRGAS 2000). This outcrop, in the Rio Bonito Formation, of Permian age, was chosen by its distinct sedimentary rocks succession as diamictite, carbonaceous pelite, and sandstone, respectively, from bottom to top. In the west face of the outcrop an area of approximately 36 square meters (6 m × 6 m) was selected for testing the developed classifier (Figure 4).

**Figure 5:**
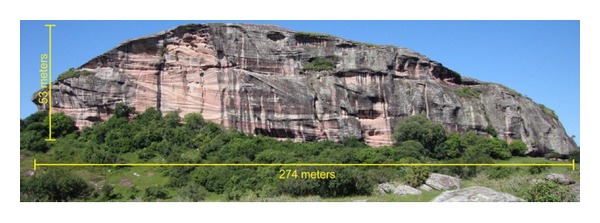
Study area B, Pedra Pintada outcrop, Pedra Pintada Alloformation. The name of this outcrop has its origin due to the different tones of reddish stratification levels. The red color came from different concentrations of iron oxide present in the cement of the sandstones.

The study area B is located in the municipality of Caçapava do Sul, approximately 330 km from Porto Alegre with access by highway BR-290, BR-392, and RS-625. In this place the outcrop called “Pedra Pintada” is located in coordinates: Latitude 30°53′56,22′′ S and Longitude 53°21′37,17′′ W. Gr (SIRGAS 2000). This outcrop belongs to the Pedra Pintada Alloformation and consists of fine to medium sandstone, well sorted, composed of well-rounded grains with high sphericity, and structured in sets of cross-stratification up to 15 m thick [41]. In this outcrop an area with 165 meters in length was selected from the main face of the outcrop in the south side (Figure 5).

## 5. Materials and Methods

The operation of TLS equipment can be described as a simple activity but presents several details and requires specific knowledge of the purpose of use to allow a total capacity utilization of the equipment. The planning of basic features such as range, both minimum and maximum, expected spectral behavior of the targets of interest, and optimal resolution to be used is indispensable for obtaining three-dimensional outcrop data, mainly data capable of being used for spectral classification.

### 5.1. Ilris 3D Laser Scanner

The Ilris 3D terrestrial laser scanner developed by the Canadian company Optech has the following technical characteristics described in Table 1 (Optech, 2009).

### 5.2. Definition of the Work Method

The initial tests described here aimed to determine the ability of the TLS equipment available to provide viable data for spatial and spectral processing and, after reviewing the available data, the methodology of work to be used. Some rock samples were placed on a bench located approximately 6 m from the laser scanner device. These samples were surveyed with a high resolution of approximately 1 mm between the points. Among the rock samples used were granite, sandstone, basalt, carbonaceous pelite, diamictite, banded iron formation, and gabbro.

The first feature identified was the high noise, whose random pattern did not allow the correct identification of the samples. Subsequent tests proved that surveys carried out with the Ilris 3D TLS in resolutions higher than 1 cm presented noise due to the diameter of the emitted laser pulse.

The second feature highlighted in the samples survey was related to the intensity values of return from the rocks. It was expected that the samples would exhibit completely different spectral signatures, which did not happen. This situation was due to the fact that the Ilris 3D equipment has been designed for long distance surveys, up to 1200 meters on an object with 80% reflectance, which, in surveys in short distances, makes the laser pulse too intense, giving a response that cannot be used to differentiate rocks.

Works using intensity values of the laser pulse return as the element of study (i.e., [42]), in general, tend to maintain a minimum distance of approximately 60 meters between the equipment and the surveyed target to avoid overexposure of the target. This can represent a disadvantage in the case of outcrops situated in narrow canyons, caves or other places where the minimum distance from the equipment to the target is not possible.

**Figure 6:**
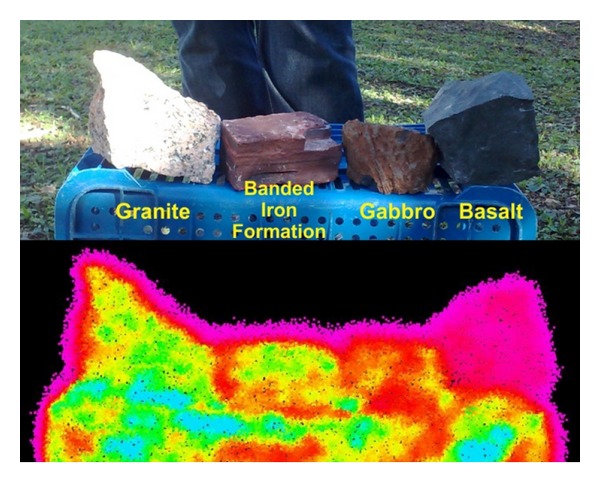
Initial survey with rock samples, 60 meters apart from the scanner. The unusual magenta points around the samples are the edge effect.

After the failure of processing the data gathered in the room, the equipment and samples were carried to the inner courtyard of the university, keeping a distance of 60 meters between the equipment and the samples. The samples were placed on a plastic holder and the survey was conducted. In this second test, since the beginning of the survey it was possible to see that at this distance, the initial results obtained attested the possibility of processing the intensity values of the samples (Figure 6).

**Figure 7:**
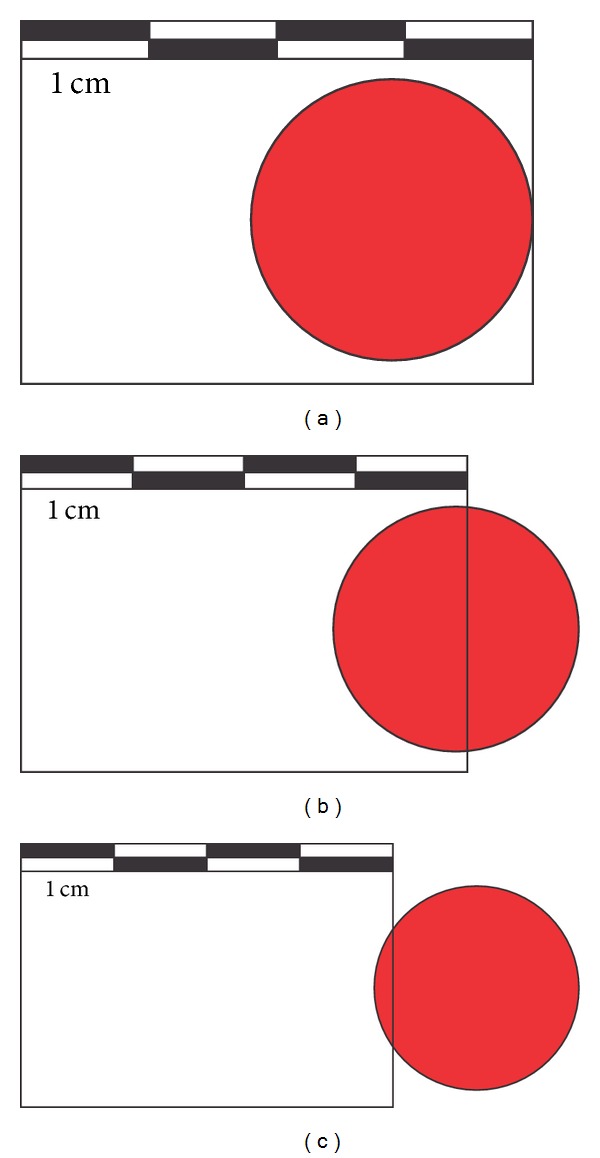
Edge effect in the targets, using the Ilris 3D divergence (2,2 cm at 100 m) and 1 cm space between the points. (a) The laser pulse in full incidence on the target. (b) The laser pulse with almost 50% incidence on the target, obtaining a second response from part of the pulse outside the target. (c) The laser pulse with only 10% of incidence in the target, obtaining an extremely low intensity primary point and a stronger second response.

Another key component identified in the survey of the samples was the presence of noise that presents a low gray level near to the minimum values found in the point cloud, around the bracket assembly and samples. This feature was due to the divergence of the laser, which, in a distance of 100 meters, covers a circle with 2,2 cm in diameter. If we consider that the spacing between points used for this survey was 1 mm and the average distance between the laser and the targets was 60 meters, we can infer that in the edges of the targets, due to the size of the laser pulse, an increasingly smaller percentage of the pulse returns, until the moment it ceases altogether, causing an area of low return (Figure 7).

### 5.3. Data Preparation

The study areas A and B had already been surveyed previously in projects conducted by the Graduate Program in Geology in Unisinos and the data were available for use in the LASERCA, which simplified the processes of study and selection of the areas.

The use of these data confirms a key feature for the surveys of point cloud acquisition, the possibility of a historical record of the outcrop and processing and interpretation even after its weathering or destruction.

The point clouds from both surveys were preprocessed in the PARSER software in order to convert the Ilris 3D raw. The intensity data of the Morro do Papaléo outcrop were exported as 16 gray levels and the Pedra Pintada outcrop with 256 gray levels of intensity. Despite the different ranges of output intensity data in the outcrops, they showed no use impossibilities for the processing of return values.

After the preprocessing of the point clouds, they were imported in the software PolyWorks (Inovx), specific software for processing data from TLS. In PolyWorks all extraneous elements were removed from the outcrop, such as vegetation, elements from anthropic action (garbage, waste materials, etc.), and any other elements that are not part of the features of the outcrop.

The survey of the study area A, Morro do Papaléo, in the city of Mariana Pimentel, was performed with an average spacing of 3 cm between the points, obtained at an average distance of 48 meters. Due to the conditions found at the site, it was not possible to install the equipment over a longer distance. However, the data collected presented the right characteristics to be processed for use of its intensity. A total of 8 stations were performed in the study area in order to obtain the entirely outcrop but were selected an area of approximately 36 square meters (6 m × 6 m) in an ideal location where they were all major facies of the outcrop on site.

The survey of the study area B, Pedra Pintada, in the city of Caçapava do Sul, was performed with an average spacing of 4 cm at an average distance of approximately 232 meters between the equipment and the face of the outcrop. The conditions found at the site allowed the installation of the equipment at a distance suitable for the collection of data which allow processing of intensity values.

At this location, the main face of the outcrop has a length of approximately 274 meters, where we selected a smaller area with a length of approximately 165 meters in order to allow greater flexibility in the experiments and tests.

The point clouds in both study areas were processed to generate histograms of the spectral distribution of rocks in the outcrops to assist in the process of choosing the number of classes to be classified.

### 5.4. K-Clouds Classification Program

Currently, the process of the intensity values of the laser scanner data is performed in conventional software for digital image processing. Aiming to spread a specific method for use of TLS in outcrops, a program was developed specifically to process point clouds by applying the clustering classification algorithm *k*-means [40, 43].

**Figure 8:**
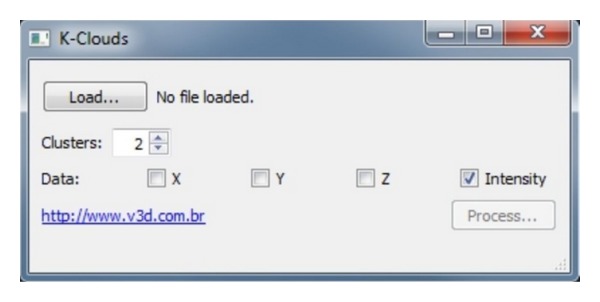
K-Clouds interface. The software allows the user to define the number of clusters linked with the intensity values (I) or spatial values (XYZ) coordinates.

This software, named K-Clouds (Figure 8), was developed both by V3D and UNISINOS. It processes the TXT or PTS file formats and perform the clustering of the intensity values considering the number of classes defined by the user.

The information is exported through a file in XYZRGB format where each class “*k*” has a unique RGB encoding, in order to ease its identification. This information can then be imported into software capable to handle point clouds. Colors are useful to enhance different spectral signatures related with different physical and chemical characteristics. They allow the identification of the rocks present in the outcrop and put in evidence characteristics that may complement the studies carried out on site.

## 6. Results and Discussion

In the initial testing phase with the samples, it became clear that the equipment used could not be operated so close to the targets with the purpose of using the intensity values of return due to its extremely strong laser pulse, masking the spectral signature of the targets. This issue has been solved by collecting data at distances close to or greater than 50 meters.

Another feature that could be proved with this survey was the relationship between data and noise found. As we improve the resolution of the survey reducing the space between the points, we add a significant amount of noise, points dislocated from the actual position of the target. Because of this feature and the difficulty of carrying out a data filtering, the selection of the proper space between the points becomes, with the use of an adequate distance between the equipment and the target, a key element for a positive or negative work outcome.

**Figure 9:**
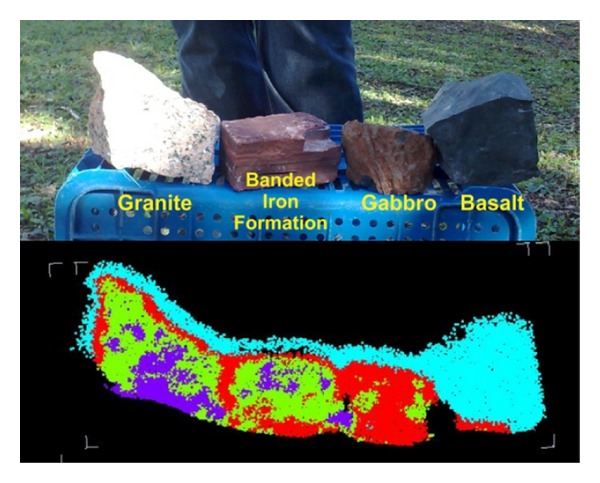
Classified point cloud from the rock samples. The basalt sample (cyan—class 1) is easily recognizable, as the edge effect above all samples, but the other samples need a specific analysis to be identified. The granite is formed by 50% of class 2 (green), 40% of class 3 (purple), and 10% of class 4 (red); the banded iron formation is formed by 70% of class 2, 15% of class 3, and 15% class 4; the gabbro is formed by 85% of class 4 and 15% class 2.

Data from the initial test were processed using the K-Clouds software and four (*k* = 4) distinct classes of rocks were selected, one class for each rock sample used. The results show a large difference between the basalt sample (located to the right) and the other samples (Figure 9).

Compared with the other samples, the basalt, due to its low reflectivity and high capacity of energy absorption due to its dark tint, presented a low return of the laser pulse emitted. These features made their identification easier by the classifier.

The external areas of the samples were subjected to the edge effect from the diameter of the laser and high resolution, providing low intensity return values. These points were wrongly placed within the same class of the basalt sample. The other samples showed minor differences regarding a specific spectral signature compared with the sample of basalt; but if we make an analysis of the amount of points from each class in the samples, we can differentiate the rocks. The granite is formed by 50% of class 2 (green), 40% of class 3 (purple), and 10% of class 4 (red); the banded iron formation is formed by 70% of class 2, 15% of class 3, and 15% of class 4; the gabbro is formed by 85% of class 4 and 15% of class 2.

**Figure 10:**
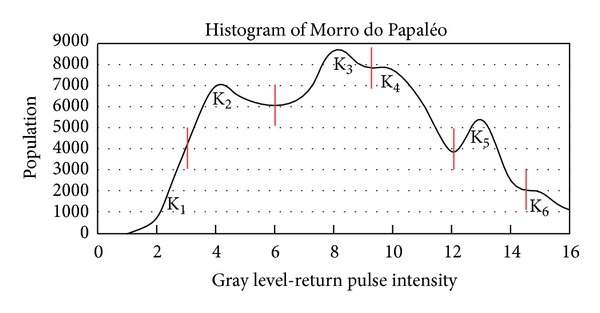
Histogram from the return intensity values from the Morro do Papaléo outcrop. The red vertical lines separate the clusters that were user-defined and applied in the K-Clouds software.

In the study area A, Morro do Papaléo outcrop, the resulting point cloud with about 51,000 points was processed to generate a histogram of the spectral distribution from the rocks available in the outcrop (Figure 10).

**Figure 11:**
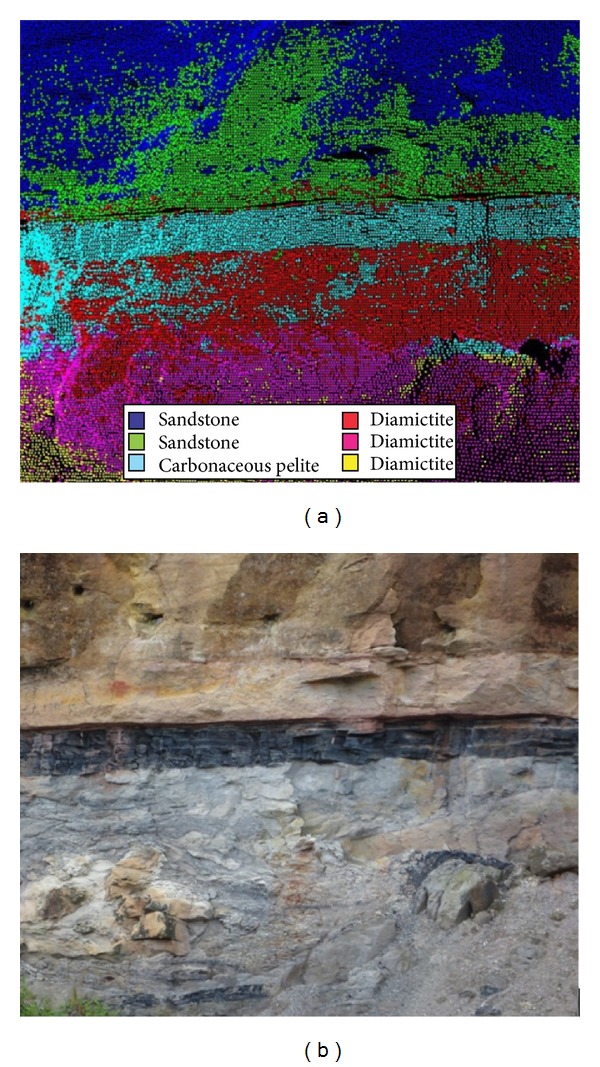
Comparison between the classified point cloud (a) and the reference image from the outcrop (b).

After the histogram analysis, the point clouds were classified into six classes of intensity return (*k* = 6) based on the spectral distribution of the intensity data (Figure 11).

In a direct visual comparison of the classified point cloud with the reference image of the site, it can be concluded that the classifier was able to identify and separate the carbonaceous pelite layer in the central portion of the outcrop (cyan). Above the carbonaceous pelite layer in the upper portion of the outcrop, the existing sandstone was separated into two classes, the colors dark blue and green. The parts placed in the dark blue exhibit a tendency to be sandstone with higher degree of weathering, while the parts placed in the green tend to be sandstone with lower degree of weathering.

At the bottom of the carbonaceous pelite, the program identified three distinct features in the diamictite, which has a tendency to be a less weathered rock near the carbonaceous pelite (red) and a more weathered rock near the base layer (magenta and yellow). It is also evident that, in the lower right portion of the study area, a small block of carbonaceous pelite detached from the main layer which was successfully identified (cyan).

**Figure 12:**
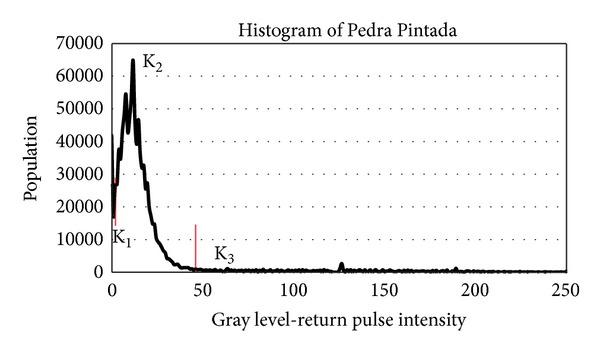
Histogram from the return intensity values from the Pedra Pintada outcrop. The red vertical lines separate the clusters that were user-defined and applied in the K-Clouds software.

In the study area B, the Pedra Pintada outcrop, with approximately 2.6 million points, was processed in the same manner as the study area A to generate a histogram of the spectral distribution of the existing rocks in the outcrop (Figure 12).

**Figure 13:**
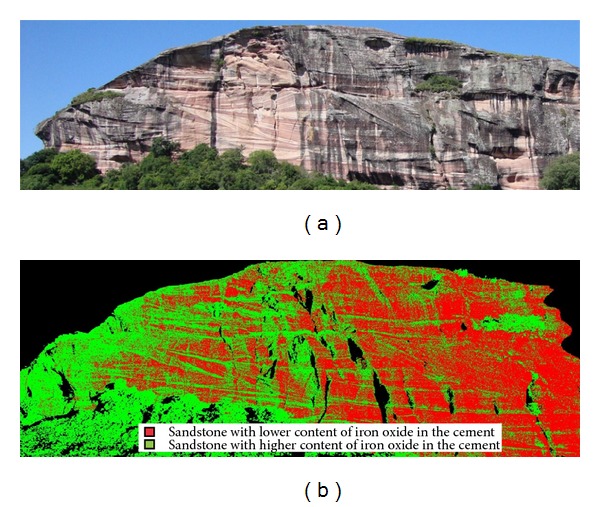
Comparison between the classified point cloud (b) and the reference image from the outcrop (a).

After the histogram analysis, it was defined that the point cloud should be classified into three classes of intensity return (*k* = 3) based on the spectral distribution of the intensity data (Figure 13). In both cases, along with the data from the histogram, a photo from the site assisted in determining the number of classes to be used.

The use of only three classes of information in this outcrop, in addition to the analysis of the histogram, is based on the separation of the different areas from the outcrop concerning the iron oxide concentration present, which is clearly characterized by the different levels of reddish hue present in the outcrop.

In the classified point cloud it is possible to identify the depositional structures, enhanced by greater or lesser concentration of iron oxide as cement. The green points refer to higher concentrations of iron oxide and the red area refers to lower concentrations of this compound in the cement of the stone.

Another factor to consider is that due to the wavelength of 1535 nanometers from the Ilris 3D, in the mid-infrared range, there is a small penetration of the laser in areas where the rock is affected by lichens or thin weathering crusts. Therefore, the spectral classification is not affected by such elements. The comparison of the photo with the spectral classification clearly shows that the sedimentary structures of the outcrop are visible even in areas with lower quality of exposure.

These type of features are very common in places with high temperature and humidity like those regions where tests were performed. To improve data acquisition procedures and processing techniques are fundamental to better represent the outcrops as digital models.

## 7. Conclusions

The use of terrestrial laser scanner for applications in geology and Digital Outcrop Modeling is a technological trend that increasingly sees its place among the various classical geologic techniques already established. Modern equipment, with longer range, higher quality, better spatial accuracy of the data, and reduced time for collection of data make the use of terrestrial laser scanner a viable and reliable tool in the execution of works.

The classification of spectral patterns of intensity from the laser pulse return for geological applications is an area that needs more developments and specific research aimed to establish and consolidate work methods. In this context, it is clear that the development of the K-Clouds software, through the use of *k*-means cluster classifier, provides a simple and practical tool to be inserted within the workflow geology professionals. However, other areas that use data from terrestrial laser scanner can use these methods.

The application of this program to classify the intensity values of point clouds from outcrops presented consistent results, being able to identify differences between several rocks presented in outcrops or even changes in chemical composition related to the presence of cement with iron oxide found in specific levels of the Pedra Pintada outcrop.

This algorithm is suitable for this type of application, being capable to distinguish different types of rocks (Figures 9 and 11) or a same rock with different chemical characteristic (Figures 11 and 13).

One of the improvements planned for the program is the use of other classifiers in addition to the *k*-means. They will provide new results for different rocks, such as carbonates and evaporites. Another element to be developed in the user interface is the automatic display of the histogram from the intensity of returned laser pulse in order to ease the user understanding and simplify the process of choosing the correct number of user-defined classes to improve the results obtained by the classifier.

## Figures and Tables

**Table 1:** Ilris 3D terrestrial laser scanner technical characteristics.

Range	Up to 1200 m
Minimum range	3 meters
Linear accuracy	7 mm at 100 m
Angular accuracy	8 mm at 100 m
Beam diameter	2.2 cm at 100 m
Laser repetition rate	2000 points per second
Laser class	Class 1 (eye safe)
Wavelength	1535 nm (infrared)
Field of view	40° × 40°
Digital camera	Integrated, 3.1 M pixel
